# Prevalence and management of mental health comorbidities in a German cohort of patients with Ehlers-Danlos syndromes and a generalized hypermobility spectrum disorder

**DOI:** 10.1186/s13023-026-04242-4

**Published:** 2026-02-03

**Authors:** Michaela Henning, Marie Hock, Arim Shukri, Vanessa Löw, Nikolaus Kernich, Jonas Rauterberg, Henning Klapproth, Iliana Tantcheva-Poór

**Affiliations:** 1 Department of Psychosomatics and Psychotherapy, University Hospital Cologne, University of Cologne, Kerpener Strasse 62, 50937 Cologne, Germany; 2 Department of Dermatology and Venereology, University Hospital Cologne, University of Cologne, Kerpener Strasse 62, 50937 Cologne, Germany; 3 Institute of Health Economics and Clinical Epidemiology, University Hospital Cologne, Kerpener Strasse 62, 50937 Cologne, Germany; 4 Department of Anesthesiology and Intensive Care Medicine, University Hospital of Cologne, Kerpener Strasse 62, 50937 Cologne, Germany; 5 Department of Orthopedic and Trauma Surgery, University Hospital of Cologne, University of Cologne, Kerpener Strasse 62, 50937 Cologne, Germany

**Keywords:** Ehlers-Danlos syndromes, Hypermobility spectrum disorder, Generalized hypermobility, Depression, Anxiety, Mental health disorders, Posttraumatic stress disorder, Eating disorders, Psychotherapy, Antidepressants

## Abstract

**Objective:**

The objective of this study was to examine mental health comorbidities and their management in individuals living in Germany with generalized symptomatic hypermobility, including Ehlers-Danlos Syndromes (EDS) and generalized hypermobility spectrum disorder (G-HSD).

**Methods:**

We conducted a descriptive cross-sectional study at the EDS outpatient service, University Hospital of Cologne, Germany. A standardized self-report form was sent to all adults who were diagnosed with hypermobile EDS (hEDS), classical EDS (cEDS), classical-like EDS (clEDS) or G-HSD from December 2021 until May 2023. Participants completed an ad-hoc standardized, pseudonymized paper-based self- report form developed ad hoc for this study as along with the following validated instruments: the PHQ-9 (Personal Health Questionnaire with 9 items) and DASS (Depression Anxiety Stress Scales) regarding depression, stress, and anxiety, and the corresponding modules of the PHQ-D (Personal Health Questionnaire, German version) regarding eating disorders and alcohol abuse.

**Results:**

A total of 132 participants were included, of whom 99 completed the questionnaire. Overall, 85% of respondents reported experiencing a moderate to severe mental burden due to EDS/G-HSD. Self-reported lifetime prevalence of mental health diagnoses was 58.6%, with depression, posttraumatic stress disorder (PTSD) and anxiety disorders being the most common ones. 27.3% indicated at least two mental health disorders. The prevalence of symptom severity scores above the clinical cut-off of depression was 60.2% (according to the PHQ-9) and that of anxiety disorders was 45.4% (according to the DASS). Despite 68.7% of participants having received psychotherapy, our findings suggest that mental health conditions were probably underdiagnosed and likely undertreated in our patients.

**Conclusion:**

This is the first study examining mental health comorbidities in patients with EDS/G-HSD living in Germany. It adds to the growing body of evidence indicating that mental health disorders, particularly depression and anxiety, are highly prevalent in EDS, regardless of the subtype. Our survey also provides increased prevalence data for PTSD and eating disorders as well as data on the use of psychotherapy and antidepressants in EDS/G-HSD. There is a critical need for improved diagnostic pathways and treatment strategies that prioritize a multidisciplinary biopsychosocial approach in the care of these patients.

**Supplementary Information:**

The online version contains supplementary material available at 10.1186/s13023-026-04242-4.

## Background

The Ehlers-Danlos syndromes (EDS) represent a group of rare inherited connective tissue disorders clinically characterized by skin hyperelasticity, joint hypermobility and tissue fragility [1, 2]. Based on the combination of clinical criteria and a molecular confirmation (if possible), the current 2017 EDS classification recognizes 13 types [1]. For example, patients with *classical EDS* (cEDS) and *classical-like EDS* (clEDS) share similar phenotypes with generalized symptomatic hypermobility and distinctive cutaneous features such as soft, hyperextensible skin and easy bruising. In addition, patients with cEDS show wide atrophic scarring. cEDS is due to mutations in *COL5A1*,* COL5A2* and rarely *COL1A*, while clEDS has been linked to genetic defects in *TNXB* and *AEBP1* [3, 4]. Generalized symptomatic hypermobility is also a major presentation of the most common *hypermobile EDS* (hEDS), the diagnosis of which relies on clinical criteria as it remains molecularly unexplained yet [5]. Patients with generalized hypermobility (gHM) and musculoskeletal complaints who do not fulfill the criteria for hEDS or other connective tissue disorders are diagnosed with a generalised “*Hypermobility spectrum disorder”* (G-HSD). Since its introduction in 2017, G-HSD has been increasingly considered a phenotypic continuum with hEDS [6–8] and recent studies suggest that h-EDS/G-HSD are more common than initially estimated [9, 10].

About two-thirds of the patients with hEDS and G-HSD suffer from multiple comorbidities, including chronic pain, functional gastrointestinal and urogenital complaints, vegetative dysautonomia, mast cell activation syndrome and fatigue [11, 12]. A significant association of hEDS/G-HSD with various mental health disorders such as anxiety, mood, eating and sleep disorders has been repeatedly reported [9, 13–17]. In a clinical study, patients with panic disorder and agoraphobia were 16 times more likely to suffer from a “joint hypermobility syndrome” (JHS) than control patients with other mental health or somatic illnesses [18]. There is a well-established association among mental health disorders, chronic pain and gastrointestinal dysfunction in hEDS and G-HSD, suggesting the need for multimodal therapeutic settings [15, 19]. However, there is only limited evidence regarding the efficacy of psychotherapeutic interventions in hEDS [20, 21] and little is known about the psychopathological aspects of the monogenetic types.

Currently, there is no information on mental health comorbidities in patients living in Germany with EDS/G-HSD as a national center and registry are missing. In 2020, we established a weekly Ehlers-Danlos dermatologic-orthopedic service at the Cologne University Hospital. To better understand our patients’ needs, we conducted a prospective survey including all adults who were diagnosed with hEDS, cEDS, clEDS and G-HSD. Here, we present our results regarding the respondents’ mental health comorbidities and their utilization of psychotherapy and antidepressant medication.

## Materials and methods

### Study design and study population

We conducted a cross-sectional descriptive study at the EDS outpatient service of the University Hospital of Cologne, Germany. The survey was approved by its ethics committee in accordance with the Declaration of Helsinki (registered as DRKS00024554).

Data were collected from December 2021 until May 2023. Inclusion criteria comprised an age of ≥ 18 years, symptomatic gHM (including G-HSD, hEDS, cEDS and clEDS), informed consent for study participation and adequate understanding of the German language. gHM was defined according to the 2017 EDS criteria as having a Beighton score of ≥ 5 for pubertal men and women up to the age of 50, and a Beighton score of ≥ 4 for women over 50 years old [1, 20]. In addition, all patients had musculoskeletal complaints in the context of a so-called “symptomatic hypermobility” [20]. An unexplained connective tissue disease, an unlisted Ehlers-Danlos subtype and/or a cognitive impairment belonged to the exclusion criteria.

After providing written informed consent, participants completed a standardized, pseudonymized paper-based self-report form developed ad hoc for this study and reviewed by domain experts to ensure content validity, as well as validated instruments, including the German versions of the Patient Health Questionnaire with 9 items (PHQ-9), the Depression Anxiety Stress Scales (DASS), and the Patient Health Questionnaire (PHQ-D). All questionnaires were administered under standardized conditions using a paper-and-pencil format.

### Outcomes

The primary endpoint of our survey was to determine the prevalence of mental health comorbidities in patients diagnosed with G-HSD, hEDS, cEDS, clEDS by assessing scores on the PHQ-9, DASS, and PHQ-D. In addition, we examined the lifetime prevalence of mental health disorders reported by participants, their burden of disease and health care utilization. Secondary outcomes were the use of psychotherapy and antidepressant medication in our cohort.

### Instruments

#### Ad-hoc study specific questionnaire

An ad-hoc study specific self-report questionnaire was used to collect information pertaining to the patients’ socio-demographic data, diagnostic journey, current impairing physical and mental health symptoms, current burden of disease and health care utilization. This instrument was developed by a multidisciplinary team comprising dermatologists, experts for psychosomatic medicine, pain specialists and orthopedists to assess EDS/HSD-specific clinical features not covered by standard psychometric tool. The mental health impact of EDS was measured using a 4-point Likert scale from 1 (no impact) to 4 (severe negative impact). Prevalence data for PTSD and other mental health diagnoses were based on self-reporting of prior formal diagnoses by a clinician. Further information on the study-specific self-assessment questionnaire.can be found in Supplement 1.

#### PHQ-9

The 9-item depression scale assesses the severity of the depressive symptoms, in which response options range on a 4-point Likert scale from 0 (not at all) to 3 (nearly every day) and the numerical values are added up to a total score (0 to 27). Different levels of severity can be assessed: ‘minimal’ (0–4 points), ‘mild’ (5–9 points), ‘moderate’ (10–14 points), ‘moderately severe’ (15–19 points) and ‘severe’ (20–27 points) depression. A cut-off score of ≥ 10 has a sensitivity of 88% and a specificity of 88% for major depression [22].

#### DASS

The short DASS version [23, 24] is included in the pain questionnaire of the German Pain Society (DSF) and is used to measure depression, anxiety and stress without distorting the results by excluding somatic items. The questionnaire consists of 21 questions and respondents rate the items on a four-point Likert scale (0–3) (from 0 = “did not apply to me at all” to 3 = “applied to me very much or most of the time”). A threshold value of ≥ 10 is utilized to identify an elevated probability of depressive disorder and stress, whereas a value of ≥ 6 is used to indicate an increased likelihood of anxiety [25]. The questionnaire also covers pain medication.

#### PHQ-D

The German version [26–28] represents a multiple-choice self-report with high reliability and validity for screening, diagnosing, assessing severity, and measuring the course of eight mental health disorders based on the Diagnostic and Statistical Manual of Mental Disorders (DSM) [28]. In the present study we only used the modules for assessing eating disorders (bulimia nervosa and binge eating disorder) and alcohol abuse.

All psychometric instruments (PHQ-9, DASS, and PHQ-D) were administered in their validated German version.

### Statistical analysis

A detailed overview of the sample size calculation is provided in the Supplement. Descriptive analysis of all socio-demographic and clinical variables was analyzed for the entire sample and for the two subgroups: monogenetic EDS (cEDS/clEDS) and hEDS/G-HSD. In concordance with previous studies, hEDS and G-HSD were combined for analysis as they probably represent a part of the same clinical spectrum without a genetic test yet [29]. Categorical variables are reported as absolute and relative frequencies, while continuous variables are presented as means and standard deviations. Total prevalences were calculated with 95% confidence intervals (CIs), using the Clopper-Pearson method for categorical variables and bootstrapping (1,000 samples) for continuous variables, the latter chosen to account for parameter uncertainty and the small sample size. To account for multiple testing, differences between monogenetic EDS and hEDS/G-HSD were assessed only for selected categorical variables (Use of antidepressants medication) using Fisher’s Exact Test, and for selected continuous variables (PHQ-9, DASS subscales) using the Mann-Whitney U test. Data were analyzed using IBM^®^ SPSS^®^ Statistics for macOS, version 30.0.0.0 (Build 172) (IBM Corp: Armonk, New York). For all statistical tests, p-values < 0.05 were considered statistically significant.

## Results

### Demographic and clinical characteristics

Of 132 participants, 99 completed the questionnaire (response rate of 75.0%). Most of them were diagnosed with hEDS/G-HSD (80.8%) and 19.2% had cEDS/clEDS. The mean age was 35.9 ± 12.8 years, with a female predominance (87.9%) and 76.8% reported living arrangements with others. No significant differences were observed between the two subgroups (monogenetic EDS [cEDS/clEDS] and hEDS/G-HSD) regarding sex, age and level of education. Table 1 describes the characteristics of participants in detail.

**Table 1:** Demographic and clinical characteristics

Characteristics	Total	Monogenetic	hEDS/HSD
	*n* (%)	*n* (%)	*n* (%)
**EDS diagnosis**	99 (100)		
**Monogenetic**	19 (19.2)		
Classical EDS	16 (16.2)		
Classical-like EDS	3 (3.0)		
**hEDS/HSD**	80 (80.8)		
hEDS	27 (27.3)		
HSD	53 (53.5)		
**Age**			
Years, mean (SD)	35.9 (12.8)	36.4 (16.3)	36.0 (12.0)
**Gender**			
Female	87 (87.9)	16 (84.2)	71 (88.8)
Male	10 (10.1)	3 (15.8)	7 (8.8)
Divers	2 (2.0)	-	2 (2.5)
**Education**			
Lower secondary education	23 (23.2)	6 (31.6)	17 (21.3)
Upper secondary education/ General university entrance qualification	47 (47.5)	9 (47.4)	38 (47.5)
Bachelor or equivalent	29 (29.3)	4 (21.1)	25 (31.3)
**Marital status**			
Single	54 (54.5)	12 (63.2)	42 (52.5)
Married/ registered partnership	38 (38.4)	6 (31.6)	32 (40.0)
Divorced	6 (6.1)	-	6 (7.5)
Widowed	1 (1.0)	1 (5.3)	-
**Living Arrangements**			
Alone	23 (23.2)	2 (10.5)	21 (26.3)
With others	76 (76.8)	17 (89.5)	59 (73.8)

Note: hEDS, hypermobile Ehlers-Danlos syndrome; HSD, hypermobility spectrum disorder; SD, standard deviation

### Symptom severity scores of mental health disorders

As shown in Table 2, the PHQ-9 indicated elevated scores for depression, meeting the cut-off point of ≥ 10 in 59 patients (60.2%) with a mean score of 11.0 ± 5.5. Depression severity was categorized as mild (30.6%), moderate (32.7%), moderately severe (21.4%) and severe (6.1%). A total of 44 patients (45.4%) met the anxiety threshold on the DASS, 28 patients (28.9%) scored above the depression threshold, and 40 patients (41.1%) reached the stress threshold. The PHQ-D analysis indicated the presence of any form of eating disorders (bulimia nervosa or binge eating disorders) in five patients (5.2%), while alcohol abuse was identified in two patients (2.1%). No significant differences in PHQ-9, DASS or PHQ-D results were found between subgroups.

**Table 2:** PHQ-9, DASS, PHQ-D questionnaire assessment at baseline

Parameter	Total (%)	95% CI	Monogenetic	hEDS/HSD
	*n* (%)		*n* (%)	*n* (%)
**PHQ-9 Depression Scale**	*n* = 98*		*n* = 19	*n* = 79
PHQ-9 ^a^, Cut off ≥ 10	59 (60.2)	[49.8, 70.0]	9 (47.4)	50 (63.3)
PHQ-9, Score, mean (SD)	11.0 (5.5)	[10.0, 12.1]	9.2 (5.7)	11.5 (5.4)
Level of Depression Severity				
Minimal, <5	9 (9.2)	[4.3, 16.7]	4 (21.1)	5 (6.3)
Mild, 5–9	30 (30.6)	[21.7, 40.7]	6 (31.6)	24 (30.4)
Moderate, 10–14	32 (32.7)	[23.5, 42.9]	6 (31.6)	26 (32.9)
Moderately severe, 15–19	21 (21.4)	[13.8, 30.9]	2 (10.5)	19 (24.1)
Severe, ≥ 20	6 (6.1)	[2.3, 12.9]	1 (5.3)	5 (6.3)
**DASS subscales**	*n* = 97^*^		*n* = 19	*n* = 78
DASS-Depression above threshold (≥ 10)	28 (28.9)	[20.1, 39.0]	4 (21.1)	24 (30.8)
DASS-Depression, Score, mean (SD)	6.7 (5.1)	[5.7, 7.8]	6.4 (4.6)	6.7 (5.3)
DASS Anxiety above threshold (≥ 6)	44 (45.4)	[35.2, 55.8]	8 (42.1)	36 (46.2)
DASS-Anxiety, Score, mean (SD)	6.3 (4.8)	[5.3, 7.3]	6.0 (4.6)	6.4 (4.8
DASS-Stress above threshold (≥ 10)	40 (41.2)	[31.3, 51.7]	6 (31.6)	34 (43.6)
DASS-Stress, Score, mean (SD)	8.4 (4.9)	[7.5, 9.4]	7.7 (5.6)	8.6 (4.7)
**PHQ-D**	*n* = 97*		*n* = 19	*n* = 78
Eating Disorders	5 (5.2)	[1.7, 11.6]	1 (5.3)	4 (5.1)
Bulimia Nervosa	1 (1.0)	[0.0, 5.6]	1 (5.3)	-
Binge Eating Disorder	4 (4.1)	[0.1, 10.2]	-	4 (5.1)
Alcohol abuse	2 (2.1)	[0.0, 7.3]	-	2 (2.6)

Note: monogenetic encompasses classical EDS and classical-like EDS; hEDS, hypermobile Ehlers-Danlos syndrome; HSD, hypermobility spectrum

PHQ-9: Patient Health Questionnaire-9, PHQ-D: Patient Health Questionnaire; CI: confidence interval. DASS: Depression Anxiety Stress Scales

^a^ PHQ-9 (Major Depression Cut off ≥ 10)

^*^Missing Data: PHQ-9 *n* = 1, DASS *n* = 2, PHQ-D *n* = 2

No significant differences between subgroups were found

### Prevalence of mental health conditions (reported by patients)

The reported prevalence of mental health diagnoses over lifetime was 58.6%: 31.3% reported one, 17.2% two and 10.1% at least three mental health diagnoses. Depression was the most common (30.3%), followed by a post-traumatic stress disorder (PTSD) (21.2%) and anxiety disorders (including both generalized and unspecified anxiety disorder) (13.1%). Table 3 summarizes the distribution of mental health comorbidities in our cohort.

**Table 3:** Mental health conditions and treatment as reported by EDS outpatients

Parameter	Total (*n* = 99)	95% CI	Monogenetic (*n* = 19)	hEDS/HSD (*n* = 80)
	*n* (%)		*n* (%)	*n* (%)
Ever diagnosed with a mental health disorder				
Any Mental health disorder	58 (58.6)	[48.2, 68.4]	9 (47.4)	49 (61.3)
1 mental health disorder	31 (31.3)	[22.4, 41.4]	6 (31.6)	25 (31.3)
2 mental health disorders	17 (17.2)	[10.3, 26.1]	1 (5.3)	16 (20.0)
At least 3 mental health disorders (range 3–5)	10 (10.1)	[5.0, 17.8]	2 (10.5)	8 (10.0)
Types of mental health disorder,				
Depression	30 (30.3)	[21.5, 40.4]	4 (21.1)	26 (32.5)
Post-traumatic stress disorder	21 (21.2)	[13.6, 30.6]	2 (10.5)	19 (23.8)
Any aniexty disorder ^a^	13 (13.1)	[7.2, 21.4]	4 (21.1)	9 (11.3)
Anxiety disorders not otherwise specified	12 (12.1)	[6.4, 20.2]	4 (21.1)	8 (10.0)
Social phobia	2 (2.0)	[0.2, 7.1]	1 (5.3)	1 (1.3)
Eating disorders	8 (8.1)	[3.6, 15.3]	3 (15.8)	5 (6.3)
Pain disorder	7 (7.1)	[2.9, 14.0]	1 (5.3)	6 (7.5)
Personality disorder	7 (7.1)	[2.9, 14.0]	-	7 (8.8)
Obsessive-compulsive disorder	2 (2.0)	[0.2, 7.1]	1 (5.3)	1 (1.3)
Somatoform disorders	3 (3.0)	[0.6, 8.6]	-	3 (3.8)
Attention-deficit (hyperactive) disorder	3 (3.0)	[0.6, 8.6]	-	3 (3.8)
Adjustment disorder	4 (4.0)	[1.1, 10.0]	-	4 (5.0)
Gender Dysphoria	1 (1.0)	[0.0, 5.5]	-	1 (1.3)
Neurotic disorder	1 (1.0)	[0.0, 5.5]	-	1 (1.3)
Schizophrenia	1 (1.0)	[0.0, 5.6]	-	1 (1.3)
Treatments				
Psychotherapy,	68 (68.7)	[58.6, 77.6]	12 (63.2)	56 (70.0)
Duration of Psychotherapy (years), mean (SD) ^b^	5.8 (6.0)	[4.5, 7.3]	6.7 (6.7)	5.6 (5.9)
Types of Psychotherapy ^c^				
Cognitive behavioral therapy	53 (53.5)	[43.2, 63.6]	11 (57.9)	42 (52.5)
Psychodynamic therapy	18 (18.2)	[11.1, 27.2]	4 (21.1)	14 (17.5)
Psychoanalysis	4 (4.0)	[1.1, 10.0]	-	4 (5.0)
Systemic therapy	1 (1.0)	[0.0, 5.5]	-	1 (1.3)
Other therapies (Gestalttherapy, Hypnotherapy)	1 (1.0)	[0.0, 5.5]	-	1 (1.3)
Psychotherapy sessions				
1–5 h	2 (2.0)	[0.2, 7.2]	-	2 (2.5)
5–24 h	5 (5.1)	[1.7, 11.5]	1 (5.3)	4 (5.1)
25–50 h	17 (17.2)	[10.4, 26.3]	4 (21.1)	13 (16.5)
at least 50 h	41 (41.4)	[31.9, 52.2]	7 (36.8)	34 (43.0)
Use of antidepressants medication ^d^	29 (30.2)	[21.3, 40.4]	3 (15.8)	26 (33.8)
current	21 (21.9)	[14.1,31.5]	3 (15.8)	18 (23.4)
previously	8 (8.3)	[3.7, 15.8]	-	8 (10.4)
Types of antidepressants ^c^				
SSRI	5 (5.2)	[1.7, 11.7]	1 (5.3)	4 (5.2)
SSNRI	12 (12.5)	[6.6, 20.8]	1 (5.3)	11 (14.3)
TCA	17 (17.7)	[10.7, 26.8]	1 (5.3)	16 (20.8)

Note: monogenetic encompasses classical EDS and classical-like EDS; hEDS, hypermobile Ehlers-Danlos syndrome; HSD, hypermobility spectrum; CI, confidence interval; SD, standard deviation; SSRI, Selective serotonin reuptake inhibitor; SSRNI, Serotonin-noradrenaline reuptake inhibitor; TCA, Tricyclic antidepressants. Diagnoses based upon anamnestic informations by the patients (multiple references possible), percentages will not add up to 100% ^a^ Anxiety Disorders include anxiety disorders not otherwise specified, social anxiety phobia. ^b^ Duration of Psychotherapy, range 1–30 years

^c^ Multiple references may occur; totals do not add up to 100%

^d^ Use of antidepressants medication, sum total of current & previously

None of the scores showed significant differences between the groups

Missing Data: Psychotherapy sessions *n* = 1, Use of antidepressants medication *n* = 3, Types of antidepressants *n* = 3

In addition, 39.8% of participants graded their mental health burden due to EDS/ G-HSD as moderate, and 45.9% as severe (values not depicted).

### Utilization of psychotherapy and antidepressant medication in our cohort

68.7% of participants reported on having received psychotherapy during their lifetime (mean duration of 5.8 ± 6.0 years); 41.4% attended more than 50 sessions. Cognitive behavioral therapy (CBT) was the most used form of psychotherapy (53.5%), followed by psychodynamic therapy (18.2%). All respondents found psychotherapy at least “slightly helpful”, over 40% rated it as “helpful” or “very helpful” (data not depicted).

A total of 29 patients (30.2%) reported on the use of antidepressant medication, with 17.7% using tricyclic antidepressants and 12.5% taking selective serotonin and norepinephrine reuptake inhibitors (SSNRIs).

Among 59 participants with PHQ-9 ≥ 10, only 21 patients (35.6%) reported on receiving antidepressants at the time of the survey. No statistically significant differences were found between the EDS subgroups in terms of self-reported mental health disorders and treatments.

## Discussion

Here, we present our data on mental health comorbidities in a German cohort of 99 patients with hEDS, cEDS, clEDS and G-HSD, who were recruited at the Cologne EDS outpatient service. Nearly 60% of participants reported on having been diagnosed with at least one mental health disorder during their lifetime, which is significantly higher than the general lifetime prevalence of mental illness in Germany (one third) [30]. Almost one-third of our patients reported two or more mental health diagnoses (up to five), with depression (30.3%), PTSD (21.2%), and anxiety disorders (13.1%) being the most common ones. The symptom severity scores exceeding a clinical cutoff of anxiety and depression measured with the validated screening tools was notably higher than the self-reported lifetime diagnosis rates, namely 60.2% for probable depression (according to the PHQ-9) and 45.4% for probable anxiety (according to the DASS). These discrepancies suggest that despite the high prevalence of mental health diagnoses in our cohort, respondents were likely still underdiagnosed and possibly undertreated with respect to their mental health comorbidities, particularly depression and anxiety disorders. It is important to note that the screening tools used in the present study refer to symptom severity scores or probable disorders when exceeding a clinical cut-off point, rather than to definitive diagnoses.

Our findings support the high mental health burden of disease experienced by patients with EDS/G-HSD. In fact, 86% of participants reported on a moderate to severe mental health burden due to EDS/G-HSD. No significant differences were identified between the subgroups, which might be also explained by the small size of the monogenetic group (*n* = 19) leading to a low statistical power. On the other hand, the lack of significant differences between the subgroups might suggest that the mental health burden in our patients is rather due to the functional hypermobility/ chronic pain phenotype than to a specific genetic defect. More research is needed to explore this further.

Symptomatic hypermobility has been repeatedly associated with various mental health disorders such as anxiety, mood, neurodevelopmental and sleep disorders [17, 31]. Our survey supports these correlations and, for the first time, provides information on mental health comorbidities in a German cohort with gHM. In our sample, 58.6% reported a lifetime diagnosis of a mental health disorder exceeding the 42.5% and 49.4% in two recent systematic chart reviews [15, 19]. This may partly reflect methodological differences, but probably also results from the longer diagnostic delays experienced by our cohort, namely on average 23 years [32] compared to 10.4 years in a survey conducted by the International Ehlers-Danlos Society [33] .

### Symptom severity scores of depression

According to the PHQ-9, 60.2% met criteria for moderate to severe depression, which aligns with the upper range of previously reported point prevalence rates (11–69%) [9, 15, 19, 34–39]. Only 30.3% reported on having been diagnosed with a depressive disorder, suggesting that depression has likely been underdiagnosed in our cohort. In comparison, previous studies have elicited lifetime prevalence rates between 25.5% and 68%, and this wide range is probably due to differences in methodology [15, 19, 40, 41].

It is noteworthy that the screening-based rate of probable depression rate in our cohort greatly exceeds the one found in the general German population, where prevalence estimates range between 8.1% and 11.6% (2008–2020) [42–44]. When comparing the results from general population studies with those from studies using screening tools, however, methodological differences should be taken into account and the differences should be interpreted with caution. Beutel et al. [43] explicitly examined the impact of the first COVID-19 wave on mental health, which was probably a co-founder in our survey, given the study timeline. Even considering that individuals with medical conditions are twice as likely to experience major depressive disorders as in the general population [45], the 60.2% prevalence in our cohort remains exceptionally high. Contributing factors may include prolonged diagnostic delays, frequent misdiagnoses, negative healthcare experiences and lack of causal treatment for EDS/G-HSD [46]. For instance, a depression rate of 38.8% (PHQ-9) was reported in patients with rheumatoid arthritis, a comparable chronic pain condition [47]. When making this comparison, it is important to be cautious, as these general population studies use different methodologies rather than screening tools alone.

### Symptom severity scores of anxiety

According to the DASS, 45.4% of participants met the threshold for an anxiety disorder. This result falls within the lower to middle range of previously reported point prevalence rates for EDS/ G-HSD, which vary between 23.6% and 74.8% [19, 36–39, 48]. Besides methodological differences, access to specialized care at our center may have contributed to some reduction in anxiety burden in our cohort. Nevertheless, the observed prevalence remains substantially higher than the 15.3% reported in the general German population, according to the DEGS1 study [42].

Chart review studies reported anxiety rates between 23.6% and 58.2% [15, 19, 38] while a recent questionnaire by the Ehlers-Danlos Society’s Global Registry found that 75% of the 505 individuals surveyed with hEDS had experienced anxiety [40]. In our survey, only 13.1% of participants reported a lifetime diagnosis of an anxiety disorder, suggesting that they have probably been underdiagnosed in this regard.

Given that 17.2% of participants reported two and 10.1% reported three or more mental health diagnoses, the high odds ratio for comorbid depression, anxiety or somatoform disorders in primary care [49] likely applies to our cohort as well. These findings are consistent with those of Hershenfeld et al., who reported at least two psychiatric comorbidities in 23% of their patients with cEDS and hEDS. conditions [19].

It appears that hypermobile individuals experience an altered sense of body awareness as a possible link between anxiety and joint hypermobility [50]. Anxiety and depression have been strongly linked to chronic pain, dysautonomia, gastrointestinal dysfunction and fatigue in EDS/ G-HSD [10, 14, 15, 19]. These disorders often present with non-specific and overlapping symptoms, which may occasionally be misinterpreted. For instance, dysautonomia and fatigue may be misdiagnosed as anxiety, panic attacks or depression, and vice versa [17, 51].

### Other mental health comorbidities

PTSD was the second most common self-reported mental health disorder in our cohort, with a prevalence of 21.2%. This figure is a self-reported lifetime diagnosis based on anamnestic information (patient recall of a prior formal diagnosis by a clinician), as no validated screening tool for PTSD was administered in this study protocol. Attention has been only recently attracted to this association with reported rates of 4.7% (*N* = 106, including cEDS and hEDS) and 33% (*N* = 503, hEDS) [19, 40]. This high prevalence might be attributed to complex medical trauma, resulting from recurrent painful medical procedures, diagnostic delays, and possible invalidation by healthcare professionals [32]. Future studies incorporating structured clinical interviews are needed to explore the link between PTSD and hypermobility more closely.

Based on the PHQ-D, 5.2% of respondents met the criteria for bulimia nervosa or binge eating disorder. This is markedly higher than the12-month prevalence of 0.9% in the general German population [52]. Furthermore, 8.1% of participants reported a lifetime diagnosis of an eating disorder, supporting prior studies that suggest an association between hypermobility and eating disorders [16, 53, 54]. EDS/G-HSD patients commonly experience gastrointestinal complaints such as motility disorders, food intolerances, temporo-mandibular disorders and dysphagia. These conditions may contribute to a dysregulated eating behavior, but they may occasionally be misdiagnosed as eating disorders [55].

In our cohort, the point prevalence of alcohol abuse according to the PHQ-D was 2.1%, which corresponds to the one in the German general population [52].

### Use of psychotherapy

58.6% of participants had a self-reported mental health diagnosis and 68.7% reported using psychotherapy at some point in their lives. This discrepancy may reflect the use of psychological interventions to treat chronic pain, which is a common complaint in individuals with EDS/G-HSD [54, 56]. Notably, the proportion of patients using psychotherapy in our cohort was markedly higher than the 24.5% of individuals in the general German population with a mental health disorder who have received psychotherapy [57]. Despite this high utilization, our data suggest that a higher proportion of participants should have been offered psychotherapy, considering the discrepancy between the reported and instrument-based prevalence rates of anxiety and depression. In a specialized Ehlers-Danlos clinic, 82.5% of patients were referred to a psychologist [58], underscoring the need for integrated mental health services.

The reported duration of psychotherapy in our survey was exceptionally long, namely 5.8 years. Over 40% of respondents had more than 50 sessions and cognitive behavioral therapy (CBT) was the most frequently used modality. All respondents rated psychotherapy as at least “slightly helpful”; 40% found it “helpful” or “very helpful”. Our findings are consistent with previous research showing that individuals with depression and a chronic somatic disorder benefit from long-term psychotherapy and generally have higher use of psychotherapy than people with depression alone [54, 55]. A recent meta-analysis of psychological interventions for EDS/G-HSD patients found that CBT was included in 4 of 10 studies (*N* = 145), leading to significant improvements in both pain control and daily functioning [20]. The therapy duration in all these studies was shorter than in our survey and as the results were largely based on case reports and retrospective data, the authors concluded that larger, controlled trials are required.

### Use of antidepressant medication

30.2% of our patients reported lifetime use of antidepressants, and 21.9% were taking antidepressants at the time of the survey. Tricyclic antidepressants were most commonly used, likely because of their additional pain modulating effect. Our findings are consistent with those of a recent Italian study involving 327 individuals with hEDS/HSD. There, 34.2% were taking antidepressants to treat both pain and mental health comorbidities [38]. Similarly, a US dataset of 2,847 adults with EDS revealed that 33.1% had a prescription claim for antidepressants over a two-year period; the rate of both antidepressant and opioid use was 20% higher than that of the matched controls [59].

Based on our prevalence data, we assume that participants were probably also pharmacologically undertreated regarding their mental health comorbidities. Only 5.2% reported being prescribed SSRIs that represent the first-line pharmacological treatment for anxiety disorders.

### Strengths and limitations

To the best of our knowledge, this is the first study to investigate mental health comorbidities in a German cohort of EDS/ G-HSD adults. Data were collected using validated psychometric instruments and an ad-hoc questionnaire. To enhance methodological reliability, the latter was created by a multidisciplinary team experienced in managing patients with EDS/ G-HSD. However, as the questionnaire relied on self-reported data, recall bias is a potential limitation of our study. National registry or insurance data were not available due to the historical neglect of hEDS/HSD and strict data protection regulations in Germany.

In addition, mental health comorbidities were assessed only at a single time point without a control group. This cross-sectional design does not allow us to draw conclusions about causality. We cannot determine if the EDS/G-HSD pathology contributes directly to mental health issues or if they are a reaction to chronic illness. The study sample was relatively small, drawn from a single specialized center with only 10% male participants. It is not representative of all EDS subtypes, and generalizability to the broader German population with EDS/ G-HSD is limited. Furthermore, the voluntary participation may have introduced a non-response bias. It is possible that non-respondents had either very low symptom burdens (feeling the survey was irrelevant) or extremely high burdens (lacking capacity to participate), which could skew the reported prevalence figures. On the other hand, patients from all over Germany were examined in the Cologne consultation service and assessed by the same medical team. High internal diagnostic validity was ensured this way, particularly for hEDS and G-HSD which rely on clinical criteria in the absence of confirmatory tests.

## Conclusion

Our study adds to the growing body of evidence indicating that mental health disorders are highly prevalent in individuals with EDS/G-HSD. It also points to increased prevalence rates of PTSD and eating disorders, and provides data on the use of psychotherapy and antidepressants in the German patients. Our results underscore the need of early screening and ongoing monitoring for mental health comorbidities in this population, as well as establishing referral pathways to specialised care.

Future studies should incorporate structured clinical interviews to validate screening results and focus on viewing treatment modalities through the lens of a biopsychosocial model of illness. Additional aspects such as body dysmorphia and body image perception should be investigated. The very high and as beneficial evaluated use of psychotherapy in our cohort should be further examined in clinical trials addressing the type and duration of psychotherapy as well as its combination with other modalities (e.g., physiotherapy, pharmacotherapy). This aligns with recent evidence on the biopsychosocial impact of hypermobility spectrum disorders, which underscores the importance of multidisciplinary approaches to care [60]. Establishing specialized interdisciplinary services is instrumental in improving the health care of patients with EDS/G-HSD.

## Supplementary Information

Below is the link to the electronic supplementary material.

Supplementary Material 1

## Data Availability

The data that support the findings of this study are available from the corresponding author upon reasonable request.
