# Supplementary material for: Prevalence and management of mental health comorbidities in a German cohort of patients with Ehlers-Danlos syndromes and a generalized hypermobility spectrum disorder
Source: Orphanet J Rare Dis. 2026 Feb 3;21:83. doi: 10.1186/s13023-026-04242-4 (PMC12958675; doi:10.1186/s13023-026-04242-4)
Supplement: Supplementary file 1 — Supplementary Material 1 [file 13023_2026_4242_MOESM1_ESM.docx]

## **Prevalence and Management of Mental Health Comorbidities in a German Cohort of Patients with Ehlers-Danlos Syndromes and a Generalized Hypermobility Spectrum Disorder**

## **Additional Information**

## **Survey distribution and response rate**

A total of 132 adult patients diagnosed with hEDS, cEDS, clEDS, or G-HSD met the inclusion criteria for the study. During their visit to the EDS Clinic in Cologne, these individuals were informed about the study verbally. Subsequently, each participant received a paper questionnaire and a written informed consent form by mail. Participants were asked to return both the completed questionnaire and the signed informed consent form.

Of the 132 eligible patients, 99 (75%) returned both documents and were included in the final analysis. Of the remaining 33 patients, 31 did not respond and two withdrew their participation.

Upon receipt, the informed consent forms were immediately separated from the questionnaires, and all survey data were pseudonymized before being entered into the study database. A reminder letter was sent to non-participants after eight weeks; however, no further follow-up took place. It is important to note that no records were kept of the total number of patients who were verbally informed about the study and declined to participate.

**Further information on the study-specific self-assessment questionnaire**

In addition to the validated instruments described in the main manuscript (PHQ-9, DASS, and PHQ-D), participants completed a pseudonymized, paper-based self-assessment questionnaire developed specifically for this study.

The specially developed questionnaire included dichotomous and categorical variables with more than two answer options, allowing participants to select multiple responses if necessary. Symptoms and diagnoses were recorded using open-ended questions, enabling respondents to list up to five complaints or up to four diagnoses. Patient satisfaction with different treatment modalities was assessed using a five-point Likert scale from 1 ("not very helpful") to 5 ("very helpful").

In addition to information on physical health, the questionnaire assessed psychological symptoms using a predefined list with multiple-choice answers (e.g., depressive symptoms, exhaustion, anxiety, recurring sleep disturbances, obsessive-compulsive symptoms, mood swings, concentration and memory difficulties) as well as an open-ended question for other complaints. Participants were also asked whether these psychological symptoms had been diagnosed or confirmed by a physician or psychotherapist, including the specialty and date of diagnosis.

The history of psychotherapeutic treatment was recorded dichotomously (yes/no). For participants with prior psychotherapy, additional information was collected regarding the underlying diagnosis, treatment durations, type of psychotherapy (e.g., cognitive behavioral therapy, psychodynamic or psychoanalytic therapy), treatment setting (individual vs. group therapy), and the total number of therapy sessions. The subjectively perceived usefulness of the psychotherapeutic treatment was assessed using the same five-point Likert scale.

(Pharmaco-)therapy was recorded in free text form and then categorized according to substance groups (analgesics, including non-opioid and opioid drugs) and type of administration ("daily" vs. "as needed").
